# Regulation of NF-κB by PML and PML-RARα

**DOI:** 10.1038/srep44539

**Published:** 2017-03-20

**Authors:** Abrar Ahmed, Xiaochun Wan, Izaskun Mitxitorena, Andrew J. Lindsay, Pier Paolo Pandolfi, Mary W. McCaffrey, Karen Keeshan, Youhai H. Chen, Ruaidhrí J. Carmody

**Affiliations:** 1 Department of Biochemistry, University College Cork, Cork, Ireland; 2 Shenzhen Institutes of Advanced Technology, Shenzhen, China; 3 GLAZgo Discovery Centre, Institute of Infection, Immunity & Inflammation, College of Medicine, Veterinary and Life Sciences, University of Glasgow, Glasgow, United Kingdom; 4 Department of Pathology, Beth Israel Deaconess Medical Centre, Harvard University, Boston, MA, 02215, USA; 5 Paul O’Gorman Leukaemia Research Centre, College of Medicine, Veterinary and Life Sciences, Institute of Cancer Sciences, University of Glasgow, United Kingdom; 6 Department of Pathology and Laboratory Medicine, University of Pennsylvania, Philadelphia, PA, 19104, USA; 7 Centre for Immunobiology, Institute of Infection, Immunity & Inflammation, College of Medicine, Veterinary and Life Sciences, University of Glasgow, Glasgow, United Kingdom

## Abstract

Promyelocytic Leukemia (PML) is a nuclear protein that forms sub-nuclear structures termed nuclear bodies associated with transcriptionally active genomic regions. PML is a tumour suppressor and regulator of cell differentiation. We demonstrate that PML promotes TNFα-induced transcriptional responses by promoting NF-κB activity. TNFα-treated PML^−/−^ cells show normal IκBα degradation and NF-κB nuclear translocation but significantly reduced NF-κB DNA binding and phosphorylation of NF-κB p65. We also demonstrate that the PML retinoic acid receptor-α (PML-RARα) oncofusion protein, which causes acute promyelocytic leukemia, inhibits TNFα induced gene expression and phosphorylation of NF-κB. This study establishes PML as an important regulator of NF-κB and demonstrates that PML-RARα dysregulates NF-κB.

The *promyelocytic leukaemia (PML*) gene was originally identified at the t(15:17) translocation breakpoint characteristic of acute promyelocytic leukaemia (APL) which leads to the formation of a PML-retinoic acid receptor-alpha (RARα) fusion protein (PML-RARα)^1^. PML is indispensable for the formation of PML nuclear bodies; sub-nuclear proteinaceous structures 0.2 to 1μm in size^2^ that are attached to the nuclear matrix and associated with chromosomal regions of high transcriptional activity^3^,^4^. PML nuclear bodies form stable and transient interactions with a large number of proteins and play an important regulatory role in apoptosis, cell cycle and transcription, which appears dependent on interacting partners^3^. PML nuclear bodies have been proposed to serve as sites of protein modification by facilitating the co-localisation of modifier proteins such as kinases, acetyl transferases, phosphatases, ubiquitin and SUMO E3 ligases, and deubiquitinases, and their substrates^5^.

Studies using PML^−/−^ mice have revealed PML as a tumour suppressor and regulator of retinoic acid-induced myeloid differentiation^6^. In addition, PML^−/−^ mice show defective innate immunity in response to *Listeria monocytogenes* infection, and develop spontaneous granulomas due to impaired macrophage function^7^. The molecular basis for the regulation of innate immunity by PML however, has not been fully elucidated, although previous studies may indicate a role for the NF-κB transcription factor^8^,^9^. NF-κB is a key regulator of the immune response and also regulates gene expression influencing cell survival, proliferation and differentiation^10^. PML is required for TNFα and DNA damage induced activation of IKKε which in turn phosphorylates NF-κB p65 at S468 to modulate the expression of a subset of NF-κB target genes^8^,^9^. However, whether this is sufficient to explain the role of PML in innate immunity is currently not clear.

The PML-RARα fusion is a major causative factor for the development of APL^1^. PML-RARα blocks the differentiation of promyelocytes which leads to the proliferation of leukaemia blasts^1^. PML-RARα delocalises PML from nuclear bodies resulting in disrupted nuclear body structure and function^11^. The PML-RARα enforced differentiation block in promyelocytes is reversed by treatment with all-trans retinoic acid (ATRA) or As_2_O_3_, both of which trigger the degradation of PML-RARα protein and lead to the restoration of PML nuclear bodies^12^.

In this study, we investigate TNFα-induced transcriptional responses in PML^−/−^ cells. Our data reveals a critical role for PML in promoting TNFα responses, in particular the transcription of NF-κB target genes. Our analyses demonstrate reduced TNFα-induced NF-κB transcriptional activity and DNA binding in the absence of PML. PML^−/−^ cells also showed significantly reduced phosphorylation of p65 at a number of sites following TNFα treatment, establishing PML as a broad regulator of NF-κB phosphorylation. Moreover, our data shows that the oncofusion PML-RARα inhibits TNFα -induced expression of NF-κB target genes and also blocks p65 phosphorylation. A bioinformatic analysis of APL transcriptomic datasets provides additional evidence for the suppression of NF-κB target genes by PML-RARα.

## Results

### PML promotes TNFα-induced gene expression

**Figure 1:**
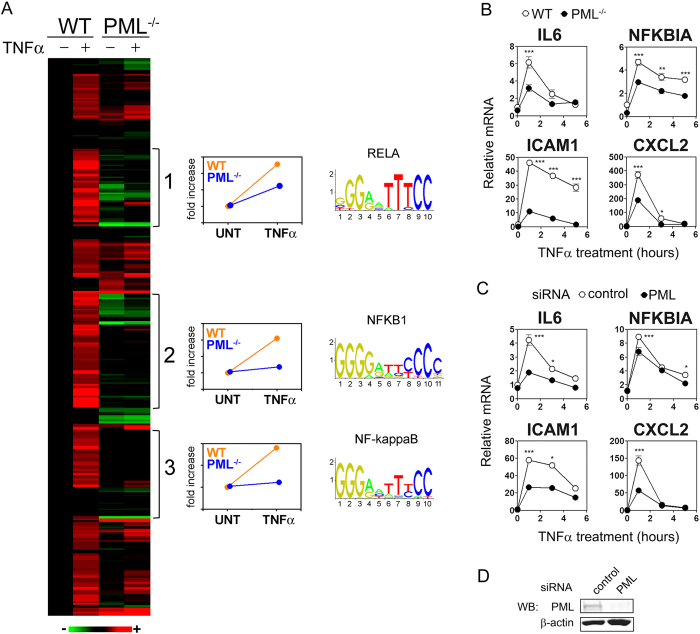
PML promotes TNFα-induced expression of NF-κB target genes. (**A**) Microarray mRNA expression data from wild type (WT) and PML^−/−^ MEFs untreated (−) or treated (+) with TNFα (10 ng/ml) for 3 hours were analysed by K-means clustering. Red represents TNFα-stimulated gene induction and green represents repression. Data is normalised on a per row basis relative to untreated WT cells. Clusters 1, 2 and 3 indicate genes expressed at reduced in PML^−/−^ cells relative to wild type (WT). The average fold induction of each cluster in WT (orange) and PML^−/−^ (blue) cells following TNFα treatment is presented. For each cluster the most significantly enriched transcription factor binding site within −2 to +2 kb of the transcriptional start sites were identified. Shown is the sequence logo for the RELA, NFKB1 and NF-kappaB matrix models in the JASPAR CORE database of transcription factor binding sites. (**B**) WT and PML^−/−^ MEFs were stimulated with TNFα (10 ng/ml) for the indicated time points and IL-6, NFKBIA, CXCL2 and ICAM1 mRNA levels assessed by QPCR. mRNA levels are expressed relative to unstimulated WT MEFs. (**C**) WT MEFs were transfected with control and PML specific siRNA prior to stimulation with TNFα (10 ng/ml) for the indicated time points. IL-6, NFKBIA, CXCL2 and ICAM1 mRNA levels were assessed by QPCR and expressed relative to unstimulated control siRNA transfected WT MEFs. (**D**) Immunoblot analysis of WT MEF cells transfected with control or PML siRNA using anti-PML and anti-β-actin antibodies. Data presented are mean ± SEM of triplicate cultures and representative of three independent experiments. Statistical significance determined by two-way ANOVA; p < 0.05 (*), p < 0.01 (**), p < 0.001 (***).

To investigate the role of PML in TNFα-induced responses we performed a microarray analysis of TNF-α treated wild type (WT) and PML^−/−^ mouse embryonic fibroblasts (MEFs). Analysis revealed three distinct clusters of TNF-α-inducible genes which were reduced in PML^−/−^ cells compared to WT cells (Fig. 1A). The genes in these clusters represent more than 50% of all genes induced by 2 fold or greater following TNFα treatment. Analysis of the promoter sequences of genes within these clusters revealed a significant over representation of NF-κB binding sites (Fig. 1A). The PML-dependent expression of selected TNFα-inducible genes (IL-6, NFKBIA, ICAM1 and CXCL2) was confirmed by QPCR in independent samples (Fig. 1B) and in WT cells transfected with PML siRNA (Fig. 1C and D). Together these data demonstrate that PML promotes TNFα-induced transcriptional responses and that NF-κB target genes are selectively regulated by PML.

### Defective NF-κB transcriptional activity in PML^−/−^ cells

**Figure 2:**
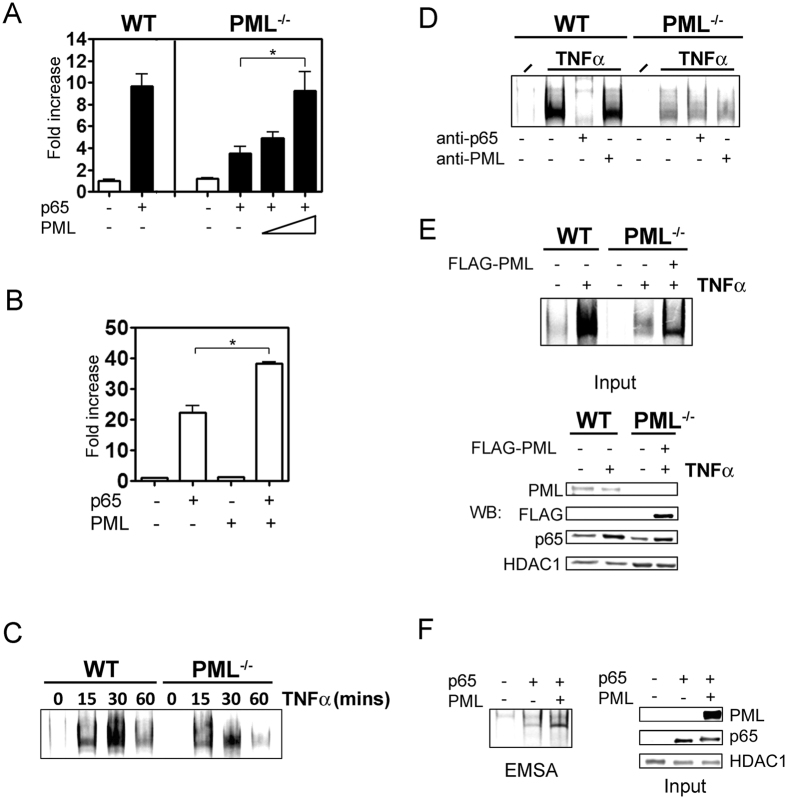
PML regulates NF-κB. (**A**) Luciferase assay using a NF-κB consensus reporter plasmid in WT or PML^−/−^ MEF cells co-transfected with an expression vector for p65 or increasing amounts of PML expression vector as indicated. Data presented are mean ± SEM of triplicate cultures and representative of three independent experiments. (**B**) Luciferase assay using NF-κB consensus reporter plasmid in HEK293T cells transfected with expression vectors for p65 and PML as indicated. Data presented are mean ± SEM of triplicate cultures and representative of three independent experiments. (**C**) WT and PML^−/−^ MEFs were stimulated with TNFα (10 ng/ml) for the indicated times analysed by EMSA using oligonucleotides for the consensus NF-κB binding site. (**D**) WT and PML^−/−^ MEFs were stimulated with TNFα (10 ng/ml) for one hour and analysed by EMSA using oligonucleotides for the consensus NF-κB binding site and antibodies against p65 and PML as indicated. (**E**) PML^−/−^ MEFs were transfected with empty vector or an expression vector for human PML and left untreated or treated with TNFα (10 ng/ml) for one hour as indicated. Untransfected WT cells treated with TNFα (10 ng/ml for one hour) or left untreated were used as controls. Nuclear lysates used in the EMSA (input) were analysed by immunoblot using the indicated antibodies. For the detection of PML a mouse specific anti-PML antibody was used. (**F**) PML^−/−^ cells were transiently transfected with PML-FLAG and p65 and analysed by EMSA using oligonucleotides for the consensus NF-κB binding site. Nuclear lysates used as input for EMSA were analysed by immunoblot using the antibodies indicated. Immunoblot for HDAC1 was used as a loading control. Data presented is representative of at least three independent experiments. Statistical significance determined by *t* test, p < 0.05 (*).

**Figure 3:**
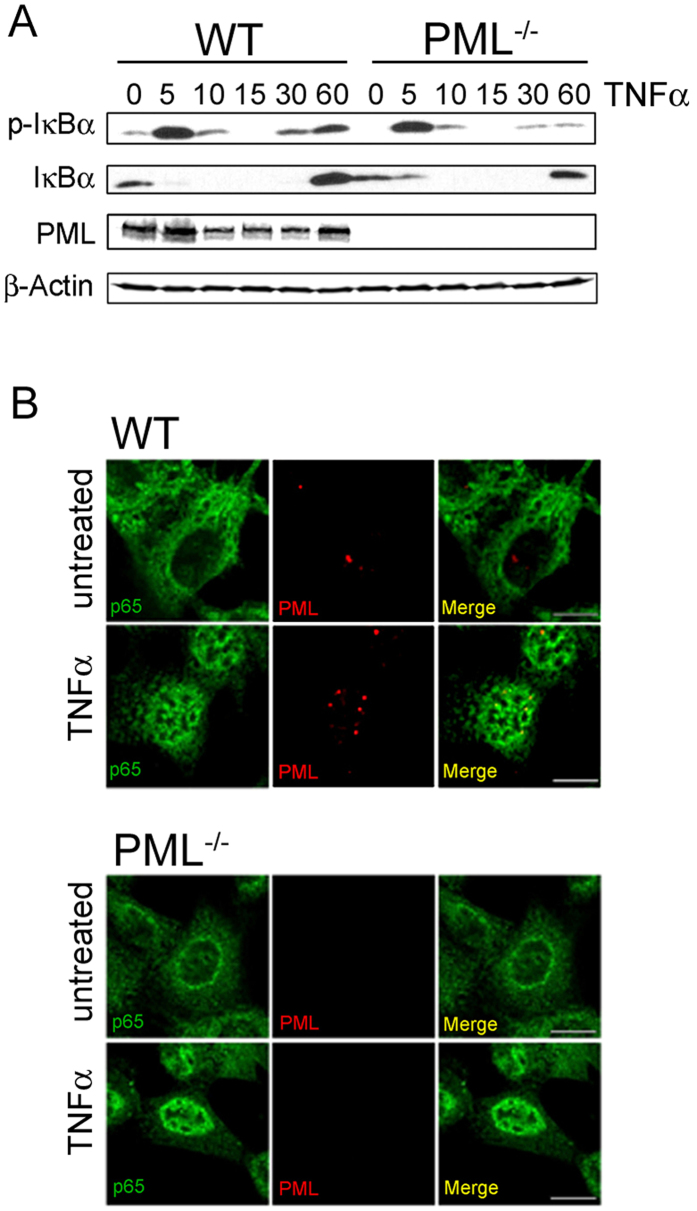
Normal NF-κB activation in PML^−/−^ cells. (**A**) WT and PML^−/−^ MEFs were stimulated with TNFα (10 ng/ml) for the indicated times and analysed by immuno-blotting with the indicated antibodies. (**B**) Nuclear translocation of p65 in TNFα treated WT and PML^−/−^ MEFs was assessed by confocal microscopy.

We next assessed p65 NF-κB transcriptional activity in WT and PML^−/−^ cells transfected with NF-κB luciferase reporter. PML^−/−^ cells demonstrated significantly lower NF-κB reporter activity relative to WT cells upon co-transfection with a p65 expression plasmid (Fig. 2A). Co-transfection of PML restored NF-κB reporter activity in PML^−/−^ cells to levels comparable WT controls (Fig. 2A). In addition, co-transfection of HEK293T cells with PML and p65 significantly enhanced NF-κB reporter activity compared to cells transfected with p65 (Fig. 2B). EMSA analysis demonstrated reduced TNFα-inducible NF-κB DNA binding in PML^−/−^ cells compared to WT controls (Fig. 2C). Supershift EMSA analysis using anti-p65 showed that PML^−/−^ cells have reduced p65 DNA binding following TNFα treatment. Furthermore, incubation of EMSA reactions with anti-PML antibodies did not lead to a detectable supershift strongly suggesting that that PML itself is not associated with NF-κB DNA complexes (Fig. 2D). Transfection of PML restored TNFα-induced NF-κB DNA binding in PML^−/−^ cells (Fig. 2E) and enhanced NF-κB DNA binding when co-transfected with p65 (Fig. 2F). TNFα treated WT and PML^−/−^ cells demonstrated equivalent phosphorylation and degradation of IκBα (Fig. 3A), and nuclear translocation of NF-κB p65 (Fig. 3B). Together these data demonstrate that PML regulates NF-κB independently of IκBα degradation and nuclear translocation.

### PML regulates NF-κB p65 phosphorylation

**Figure 4:**
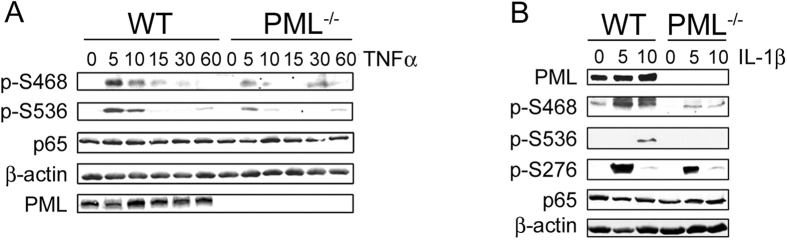
Reduced NF-κB phosphorylation in PML^−/−^ cells. WT and PML^−/−^ MEFs were stimulated with (**A**) TNFα (10 ng/ml) or (**B**) IL1β (10 ng/ml) for the indicated time (minutes) and analysed by immuno-blotting with the indicated antibodies.

Previous studies identified a role for PML in the activation of IKKε which phosphorylates p65 at S468 to inhibit the expression of genes including Icam1 and Csf2^8^,^9^. However, our data shows that PML promotes the TNFα-induced expression of both Icam1 and Csf2 (Fig. 1A) demonstrating that the regulation of NF-κB activity by PML is distinct from its role in regulating IKKε activation. Thus our data identifies a critical role for PML in promoting NF-κB activity which is not explained by IKKε mediated phosphorylation of p65. Indeed, our analysis demonstrates reduced phosphorylation of p65 at S536 in addition to S468 following TNFα treated PML^−/−^ cells (Fig. 4A), as well as reduced IL1β-induced phosphorylation of p65 at S276, S468 and S536 (Fig. 4B). We did not detect TNFα-induced phosphorylation of p65 at S276 (supplementary Figure S1). This establishes PML as a broad regulator of cytokine-induced p65 phosphorylation.

### PML-RARα inhibits TNFα responses

**Figure 5:**
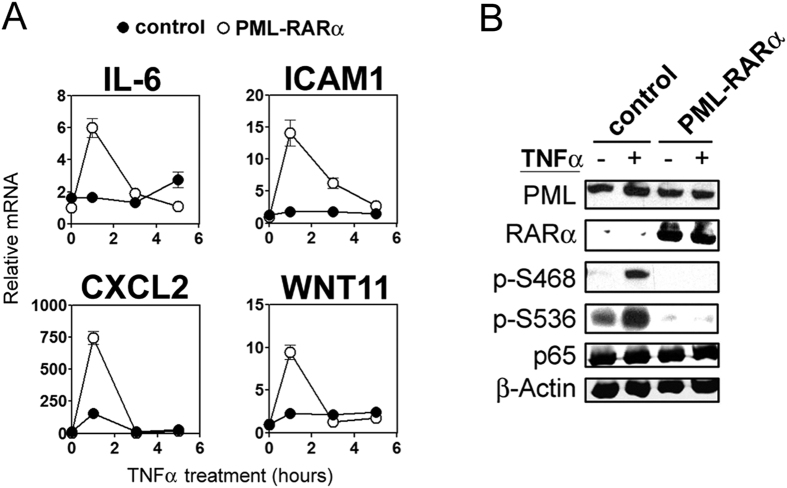
PML-RARα supresses NF-κB activity. WT MEFs were transfected with empty vector (control) or PML-RARα plasmid and were (**A**) treated with TNFα (10 ng/ml) for the indicated time prior to QPCR analysis for IL-6, ICAM1, CXCL2 and WNT11; (**B**) treated with TNFα (10 ng/ml) for 10 minutes prior to analysis by immunoblotting with the indicated antibodies.

**Figure 6:**
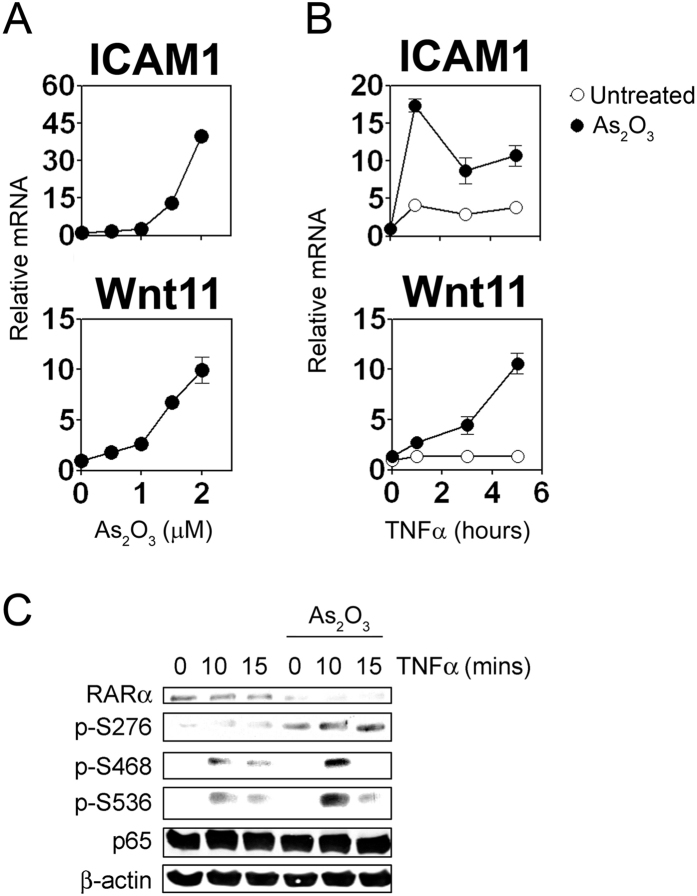
As_2_O_3_ increases TNFα induced gene expression and NF-κB phosphorylation. (**A**) NB4 cells treated with As_2_O_3_ prior to QPR analysis. (**B**) NB4 cells pre-treated with As_2_O_3_ (0.5 μM) were stimulated with TNFα before QPCR analysis or (**C**) immunoblotting with the indicated antibodies.

The PML-RARα oncofusion leads to the development of APL and dysregulated normal PML function^1^. We tested the effect of PML-RARα expression on TNFα-induced expression of NF-κB genes target genes. PML-RARα transfection in WT MEFs inhibited TNF-induced expression of IL-6, Icam1, Cxcl2 and Wnt11. Moreover, PML-RARα expression also inhibited TNFα induced phosphorylation of p65 at S468 and S536 (Fig. 5B). Thus, PML-RARα expression recapitulates the PML^−/−^ phenotype with respect to NF-κB and establishes PML-RARα as an inhibitor of NF-κB. Treatment of cells with ATRA or As_2_O_3_ induces PML-RARα degradation and differentiation of APL cells^12^. Treatment of the PML-RARα expressing APL cell line NB4 with As_2_O_3_ dose dependently increased the expression of Icam1 and Wnt11 expression (Fig. 6A) and significantly increased TNFα-induced expression compared to controls (Fig. 6B). As_2_O_3_ treatment also increased TNFα-induced phosphorylation of p65 at S276, S468 and S536 (Fig. 6C). Analysis of available transcriptomic datasets^13^ revealed an enrichment of NF-κB binding sites in genes upregulated by ATRA treatment of NB4 cells and in genes bound by PML-RARα (Table 1). Gene ontology analysis of the genes bound by PML-RARα and containing NF-κB binding sites revealed an enrichment of genes involved in haematopoiesis, immune system development and myeloid cell differentiation, suggesting that PML-RARα blockade of NF-κB activity may also contribute to the block in myeloid cell differentiation seen in APL (Table 2).

## Discussion

Here we have identified PML as a key regulator of TNFα-induced transcriptional responses through the NF-κB transcription factor. This important role for PML in regulating NF-κB transcriptional activity may also contribute to the observed immunodeficiences of PML^−/−^ mice^7^ and the phenotype of APL. Indeed, our analysis of macrophages from WT and PML^−/−^ mice shows a significant reduction of NF-κB DNA binding and reduced expression of the NF-κB target genes IL-12p40 and TNFα in LPS stimulated PML^−/−^ macrophages compared to WT controls (Supplementary Figure S2). In addition, we show that expression of the PML-RARα oncofusion protein recapitulates the PML^−/−^ phenotype and establishes PML-RARα as a negative regulator of NF-κB.

PML nuclear bodies are associated with regions of active transcription^14^,^15^ and are localised within the nucleus in a locus specific manner^14^,^16^,^17^,^18^,^19^. PML interacts with a number of proteins in the nucleus which perform a wide range of cellular functions including regulation of the cell cycle, apoptosis and DNA damage repair^20^. Our data further defines a role for PML in promoting NF-κB transcriptional activity and shows that PML acts as a broad regulator of NF-κB phosphorylation. A role for PML in the activation of IKKε has previously been described. Among other substrates, IKKε may also phosphorylate NF-κB p65 at S468 to both increase and decrease the expression of a number of NF-κB target genes^8^,^9^. Our data, however, suggests that PML plays a much broader role in regulating NF-κB activity by promoting the TNFα- and IL1β-inducible phosphorylation of a number of sites of p65 including S536 and S276 which is not dependent on IKKε activity^8^,^9^,^21^. Moreover, our analysis shows that PML promotes the expression of genes that are inhibited by IKKε phosphorylation of p65 at S468, demonstrating that the role of PML in regulating NF-κB is distinct from the regulation of IKKε activation. In contrast to the findings presented here a previous study suggested that PML negatively regulates NF-κB activity to promote apoptosis^22^. The reasons for the different findings between that study and this are unclear but our study is supported by data from PML^−/−^ MEFs and macrophages as well as our analysis of PML-RARα expression which exerts a dominant negative effect on PML.

PML nuclear bodies are sites of dynamic protein-protein interactions and have been proposed to serve as sites of protein modification in the nucleus^3^. Our data suggests that PML promotes the phosphorylation of NF-κB on at least three sites. A number of kinases of NF-κB, such as PKA, IKKε, CK2, and ATM, as well as the NF-κB phosphatase PP2a, have been demonstrated to interact with PML nuclear bodies^5^ suggesting one possible mechanism by which PML may promote NF-κB transcriptional activity. However, it is also possible that PML plays an indirect role in regulating NF-κB activity through other regulatory factors.

We have also demonstrated that PML-RARα expression blocks the transcription of TNF-α-induced NF-κB target genes and p65 phosphorylation similar to PML deficiency. These findings identify PML-RARα as a negative regulator of NF-κB activity. Our analysis of the data from the study of Martens *et al*.^13^ identified an over-representation of NF-κB binding sites in genes repressed by PML-RARα, many of which play a role in regulating myeloid cell differentiation. Previous studies have demonstrated that the inhibition of NF-κB does not block ATRA induced granulocytic differentiation of APL cells but does significantly increase ATRA-induced cell death^23^. Taken together with our data, this suggests that while the suppression of NF-κB activity by PML-RARα may potentially contribute to the differentiation block characteristic of APL, NF-κB activation is not essential to ATRA-induced granulocytic differentiation of APL cells.

## Methods

### Cell Culture, Transfection, and siRNA Knockdown

Wild type and PML^−/−^ mouse embryonic fibroblasts (MEF) and HEK293T cells were maintained in Dulbecco’s modified eagle medium (DMEM) containing 10% v/v foetal bovine serum, 2 mM glutamine and 100 U/ml penicillin/streptomycin at 37 °C and 5% CO_2_. Plasmids used were pRK5 FLAG-PMLIV (human), pCMV FLAG p65 (mouse), pGL3B NF-κB consensus luciferase reporter plasmid and pRL-TK renilla luciferase plasmid as previously described^24^. Transfection of expression plasmids was carried out using Turbofect (Fermentas) for HEK293T cells and Attractene (Qiagen) for MEF cells. Control or PML siRNA (5′-CCCAGCATATCTACTCCTTTA-3′) was transfected with Hiperfect (Qiagen).

### Gene expression analysis

RNA was extracted by RNeasy kit (Qiagen). Realtime PCR (qPCR) was performed using primer sequences as previously described^25^ and relative mRNA levels were calculated using the ΔΔCT method. Microarray analysis was performed by Beckmann Genomics and analysed using ArrayStar (DNAStar). Data are available at the NCBI Gene Expression Omnibus (GSE47828). NF-κB luciferase reporter assays were performed as previously described^24^. The detection of over-represented conserved transcription factor binding sites was performed using oPOSSUM^26^. The data set used for the identification of genes repressed by PML-RARα expression was from the study of Martens *et al*.^13^. Gene ontology analysis was performed using DAVID^27^.

### Immunoblotting, immunoprecipitations and antibodies

Whole-cell proteins were extracted using lysis buffer (50 mM Tris·HCl, pH 7.4, 1% Nonidet P-40, 0.25% SDS, 150 mM NaCl, 1 mM EDTA, with protease and phosphatase inhibitors). Anti-p65 (sc-372), anti-PML H-238 (sc-5621), anti-HDAC1 (sc-7872) purchased from Santa Cruz; anti-p65 phospho-S468 (3039S), anti-p65 phospho-S536 (3031S), anti-phospho-IκBα (9246) and anti-IκBα (4812), were from Cell Signalling Technology. Other antibodies used were anti-β-actin (Sigma), anti-hemagglutinin (HA) (Roche), anti-FLAG (Sigma), and anti-PML (mouse specific) (Millipore, 05-718).

### Immunofluorescence and confocal microscopy

Cells were fixed in 4% para-formaldehyde and permeabilised in 0.1% Triton X-100 prior to blocking in 1% BSA. Cells were incubated with primary antibodies followed by Alexa Fluor 488 and 594 conjugated secondary antibody (Invitrogen). Images were acquired on a Zeiss LSM 510 META laser scanning confocal microscope using a PlanApo 63×, 1.4 NA oil-immersion objective and a 488 nm argon and 543 nm HeNe laser lines. Images were processed in Zeiss Zen 2011 software.

### Electrophoretic mobility shift assay (EMSA)

Nuclear extracts were prepared using a Nuclear Extract kit (Active Motif). Reactions were prepared using 5 μg of nuclear extract with fluorescently labelled oligonucleotides (5′-AGT TGA GGG GAC TTT CCC AGG C-3′) (Eurofins MWG) in a 25 μl reaction volume containing 10 mM HEPES-KOH (pH7.9), 50 mM KCL, 2.5 mM MgCl2, 1 mM DTT, 10% glycerol, 1 μg DNase free bovine serum albumin and 2.5 μg poly[d(I-C)]. For super-shift assays the reactions were incubated with 1 μg of anti-p65 (sc-372) or anti-PML antibody on ice for 20 minutes before addition of the labelled oligonucleotide probe. This anti-p65 antibody inhibits p65 binding to the probe leading to a reduced EMSA band intensity corresponding to p65 DNA binding activity. Reactions were resolved on a 4% non-denaturing polyacrylamide gel.

## Additional Information

**How to cite this article:** Ahmed, A. *et al*. Regulation of NF-κB by PML and PML-RARα. *Sci. Rep.* **7**, 44539; doi: 10.1038/srep44539 (2017).

**Publisher's note:** Springer Nature remains neutral with regard to jurisdictional claims in published maps and institutional affiliations.

## Supplementary Material

Supplementary Information

## Figures and Tables

**Table 1:** Enrichment of NF-κB binding sites in genes repressed by PML-RARα identified by Martens *et al*. 13.

Factor	JASPAR ID	z-score	Fisher score
**NF-κB binding sites enriched in genes upregulated by ATRA treatment of NB4 cells**			
NFKB	MA0105.1	65.870	25.600
NF-KappaB	MA0061.1	54.991	11.949
REL	MA0101.1	36.077	7.155
RELA	MA0107.1	35.260	8.094
**NF-κB binding sites enriched in genes bound by PML-RARα.**			
NF-KappaB	MA0061.1	24.512	38.293
NFKB1	MA0105.1	22.585	32.796
REL	MA0101.1	20.515	32.875
RELA	MA0107.1	17.850	24.352

JASPR ID refers to the matrix model in the JASPAR CORE database. Sites with a z-score >10 or a Fisher score of >7 are considered significantly enriched^26^.

**Table 2:** Gene ontology (GO) analysis of genes repressed by PML-RARα^13^ and containing NF-κB binding sites.

GO term	Probability
Haematopoiesis	8.5E-7
Leukocyte activation	1.2E-6
Haematopoeic or lymphoid organ development	2.8E-6
Immune system development	5.9E-6
Lymphocyte activation	1.9E-5
Myeloid cell differentiation	2.8E-4
Leukocyte differentiation	2.1E-3
Regulation of myeloid cell differentiation	2.7E-3
Negative regulation of myeloid differentiation	7.8E-3
Lymphocyte differentiation	1.2E-2
Regulation of myeloid leukocyte differentiation	1.8E-2
Negative regulation of cell differentiation	2.9E-2
Positive regulation of myeloid cell differentiation	7.5E-2
