# Supplementary material for: Regulation of NF-κB by PML and PML-RARα
Source: Sci Rep. 2017 Mar 20;7:44539. doi: 10.1038/srep44539 (PMC5357907; doi:10.1038/srep44539)
Supplement: Supplementary Information [file srep44539-s1.pdf]

Regulation of NF-κB by PML and PML-RARα

Abrar Ahmed, Xiaochun Wan, Izaskun Mitxitorena, Andrew J. Lindsay, Pier Paolo Pandolfi,

Mary W. McCaffrey, Karen Keeshan, Youhai H. Chen, Ruaidhrí J. Carmody

Supplementary Information

Supplementary Figure S1

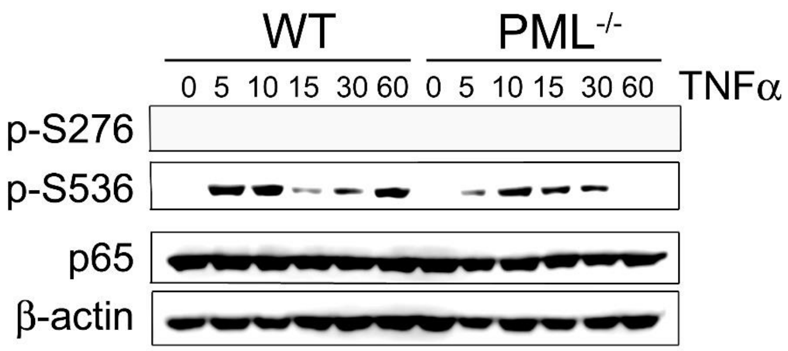

Supplementary Figure S1: WT and PML$^{-/-}$ MEFs were stimulated with TNFα (10ng/ml) for the indicated time (minutes) and analysed by immuno-blotting with antidobies for p65, phospho-S276 p65, phospho-S536 p65 and β-actin.

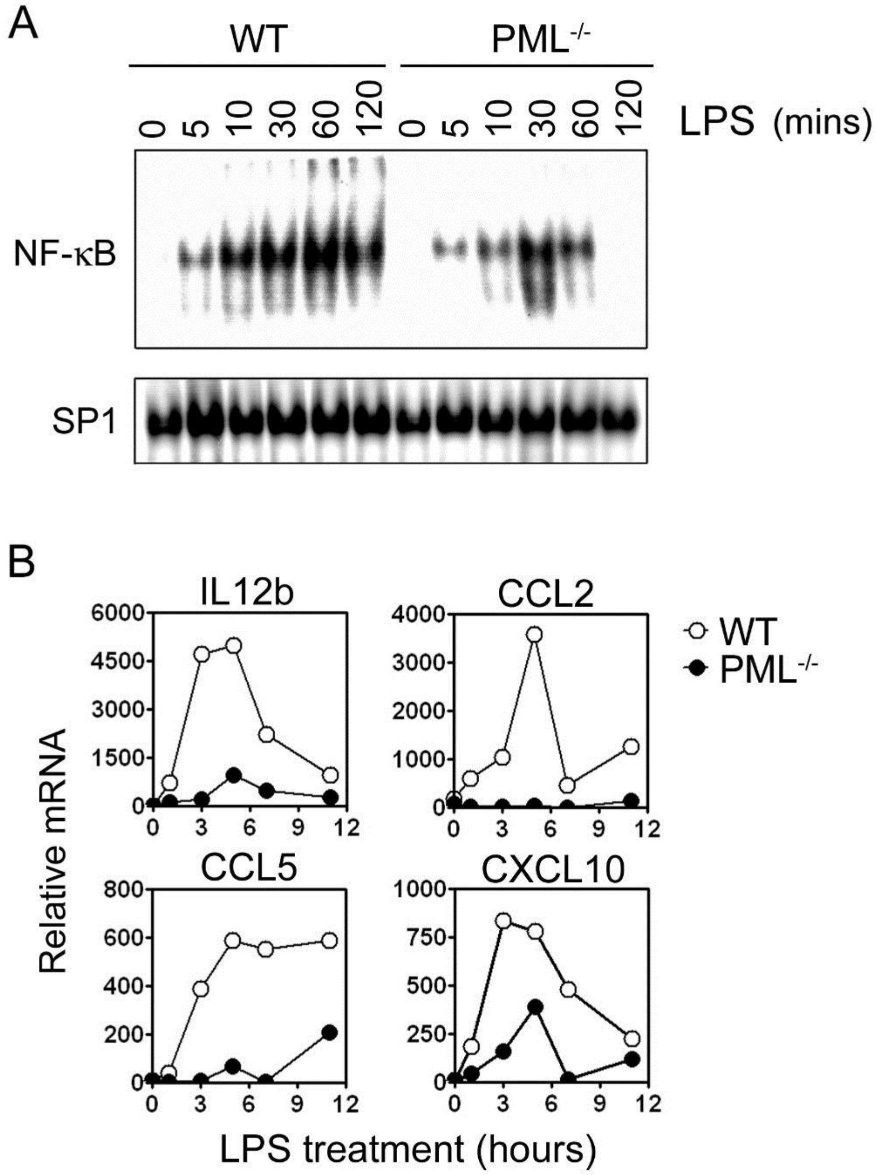

Supplementary Figure S2: (**A**) WT and *PML*$^{-/-}$ macrophages were stimulated with 100ng/ml LPS for the indicated times. Nuclear extracts were prepared and analysed by EMSA using oligonucleotides for the consensus NF-κB and Sp1 binding sites. (**B**) Real-time-PCR analysis of IL-12b, CCL2, CCL5 and CXCL10 mRNA from WT and PML$^{-/-}$ bone marrow derived macrophages treated with LPS for the indicated times.
